# Community Structure of Terrestrial Invertebrates Inhabiting a Tidal Marsh Islet in the Mediterranean Sea (Gulf of Gabes, Tunisia)

**DOI:** 10.1100/tsw.2002.159

**Published:** 2002-03-29

**Authors:** I. Colombini, L. Chelazzi, M. Fallaci

**Affiliations:** Dipartimento di Biologia Animale e Genetica Leo Pardi, Università degli Studi di Firenze, Via Romana 17, 50125 Firenze, Italy; Instituto per lo Studio degli Ecosystemi, Consiglio Nazionale delle Ricerche, Via Romana 17, 50125 Firenze, Italy

**Keywords:** arthropod community, diversity, salt marsh islet, macrotidal Mediterranean ecosystem

## Abstract

The composition of the terrestrial arthropod community of a tidal marsh islet in the Gulf of Gabes (Tunisia) was studied during two seasons (spring, autumn). The study was conducted on a small islet located in an area where the highest tidal excursions of the Mediterranean occur. Standard trapping methods (pitfall traps, mobile cages) were used to evaluate specie richness and abundance in different areas of the islet. Diversity indices were calculated for coleopterans and isopods alone. The structure of the arthropod community varied a great deal from one season to the other and differences were found when seaward areas were compared with landward ones. El Bessila presented a particularly rich beetle community whereas only few isopod species occurred. The moderately high diversity levels found for the beetle indicate the influence of the high tidal excursions in modelling the structure of the community.

## INTRODUCTION

Salt marshes are important ecosystems where a large variety of highly specialised terrestrial invertebrates inhabit. Recently Mediterranean salt marshes have dramatically reduced in size in relation to human activities therefore conservation measures are urgently needed. Although generally less studied than Western European marshes[1,2,3,4,5,6,7,8], studies on Mediterranean tidal marshes have mainly focused on species richness, abundance, and community structure of macrobenthic fauna[9,10,11] and only few studies have analysed the terrestrial faunal composition of these habitats[12,13,14,15].

The Mediterranean Sea is characterised by microtidal conditions, but as the weak tides of the currents pass Tunisia via the deep Sicily Channel these are enlarged by the gently rising floor of the Pelagean Sea and the Gulf of Gabes. As consequence these produce high tidal excursions in the entire Gulf of Gabes. The archipelago of Kneiss can be seen as the estuary of two oueds, that towards the coast widen into a huge and flat extensive delta plain with salt marshes. Within a study of low coastal sandy habitats, the islet of El Bessila was chosen because of its peculiar features. This islet cannot be considered an isolated environment in relation to its close distance with the mainland and to its connection with land during the syzygyal tides. But perhaps the most striking characteristic that models the community structure of both plant and animals of the area is the presence of the high tidal excursions. Furthermore it presents a quite pristine salt marsh vegetation cover due to protection measures since 1995. The aim of the study was to analyse the terrestrial arthropod community and to determine species richness, abundance, and diversity levels in a Mediterranean salt marsh habitat subjected to high tidal excursions.

## MATERIALS AND METHODS

### Study Area: Dzirat El Bessila (Kneiss Islets)

The Kneiss achipelago is made up of salt marshes and sebkhas intersected by tidal channel networks. The delta plain develops gently into extensive intertidal flats where several isles can be found. The most northern islet, El Bessila (34°22’N 10°19’E), has a surface of 5 km$^2$ and presents low vegetation assemblages of Sarcocornietea Fruticosae with formations of the Saginetea Maritimae in the clearings. The islet height reaches a maximum of 7 m (a.s.l.). The sediment of the intertidal area is mainly muddy but in some places it is sandy. Mean tidal excursions in this area vary from 40 to 160 cm at neap and spring tide, respectively. During low tides of spring tide, 146 km$^2$ of intertidal flats remain exposed and are mainly covered by *Zostera* sp. As consequence the seaward coasts of El Bessila are extremely sheltered and the habitat is typically salt marsh.

### Sampling Methods

The arthropod population was investigated in two different seasons (April, October 1999). To this aim samples were taken to obtain information both from a quantitative and qualitative point of view. A standard trapping system using pitfall traps along transects was used to capture animals spontaneously active at the surface. Two (A and B) out of four transects were placed on the coast of the islet looking towards the open sea. The other two transects (C and D) were situated inland, on the side of the islet looking towards the continent. These were reached by water only by the high waters of spring tides. Traps were active for 72 consecutive hours and were controlled periodically on account of the alternating high-low tides.

All transects were subdivided in three zones according to the vegetation cover. For transects A and B, zone 1 was characterised by *Arthrocnemum perenne*, *Halimione portulacoides,* and *Salicornia europaea*; zone 2 was composed of areas with clearings where assemblages with *Sphenopus divaricatus*, *Filago mareotica*, *Limonium avei*, and *Lotus* sp. were found; and zone 3 presented a halophyte scrub composed by *Limoniastrum monopetalum*, *Zygophyllum album*, *Limonium sinuatum,* and *Suaeda* sp. For transects C and D, zone 1 included plants of *Halocnemum stobilaceum* on the mudflat, zone 2 presented areas where scarce plants of *Suaeda* sp. were found, and zone 3 where a thicker vegetation cover was present.

To evaluate the macrofauna/plant ratio, mobile cages of unitary units (1 m$^3$) were used. For each transect samples were made in all zones and in both seasons. The fauna present in 1 m$^3$ of vegetation was caught and the corresponding vegetation was weighed (fresh weights).

## Statistical Analysis

Samples obtained were sorted and individuals were identified to order level. Data of Diptera caught with pitfall traps were not considered because they were probably biased in relation to the species attraction to the traps. For diversity indices only isopods and coleopterans were chosen and sorted further. The choice of these two orders was based on the consideration that for crustaceans, Amphipoda presented unidentifiable juveniles whereas Isopoda were equally represent in species numbers and were easily recognisable, and for the Insecta, Coleoptera were the most representative of this environment. For statistical analysis, data regarding transects A-B and C-D were cumulated respectively when calculating the percentage of the total number of animals caught with pitfall traps and with mobile cages.

For ecological coefficients, data from all transects were cumulated and considered in the two seasons. Relative abundance was used to provide an objective estimate of the structure of the community[16,17]. This involved estimating the abundance of individual species, as a function of the total number of individuals gathered in a particular zone or season. Species were then grouped as abundant ($A \geq 5\%$), influent ($2 \leq A < 5\%$), and recedent ($A < 2$). Fisher's[18] $\alpha$ diversity index was used and confidence limits were calculated using the standard error given in Williams'[19] monograph. To analyse the evenness of the community, Pielou's[20] evenness index through Brillouin[21] index was used instead of Shannon–Weaver index. To express the abundance of the commonest species as a fraction of the total number of individuals Simpson's[22] dominance index was calculated.

Macrofauna/plant ratio was calculated as the number of specimens per kilogram of fresh plant material.

## RESULTS

In April, 39,928 were captured with pitfall traps and 668 with mobile cages. In October, a slightly higher number of individuals were sampled (n = 43,787), of which 40,529 were captured with pitfall traps and 3,258 with mobile cages.

In April, the most represented category sampled with pitfall traps of transect A and B was the Amphipoda order with 36.20% of the total captures (n = 31,177 in April and 39,629 in October). These species belonged to the *Orchestia* genus and were identified in *O. montagui*, *O. stephenseni,* and *O. gammarella.* These were sampled exclusively in zone 1. During autumn season there was a great increase of amphipods reaching 85.15% of captures. The second most represented category in spring was the Collembola order (23.75%) that entirely disappeared from the samples in autumn months. These two orders were followed by the Formicidae family that mainly occupied the clearings of zones 2 and 3 and were present with 19.81 and 11.08% of captures in April and October, respectively. Other categories that need mentioning are the Isopoda (4.50%), the Araneae (2.31%), and the Coleoptera Staphylinidae (6.99%) that occurred principally during spring. Considering the mobile cages of transect A and B in April, the most representative category belonged the Coleoptera Carabidae (33.26% of the total captures n = 490). This family occupied all sampled zones but was mainly found in zone 2. During autumn its presence was greatly reduced and 93.58% of the total captures (n = 3,212) was represented by the Amphipoda. In spring, other categories such as Isopoda and Formicidae reached 21.02 and 17.14% of captures and occupied zone 1 and all zones, respectively. Other forms such as Araneae, Pseudoscorpiones, and Diptera were found in 4.28, 2.44, and 2.44% of total captures.

Pitfall traps of transect C and D captured 8,751 arthropods in April. Of these, 73.13% were represented by Collembola (mainly in zone 2), 11.88% by Formicidae (mainly in zone 3), whereas Coleoptera Carabidae and Staphylinidae (mainly in zone 1) were present with 4.26 and 2.98% of captures. In October (n = 900) the Formicidae increased to 49.55% whereas Araneae to 18.66%. Of the Coleoptera, the Cicindelidae family was the most abundant in zone 1 and reached

14.55% of total captures whereas Carabidae and Staphylinidae decreased to 2.77 and 1.66%, respectively. With mobile cages of transect C and D, a total number of 178 and 46 individuals were captured in April and in October. The most represented categories in April were Carabidae (51.68%), Hemiptera (13.48%), Araneae (8.89%), and Formicidae (5.05%). In October Araneae, Formicidae, Thysanoptera, Diptera, and Pseudoscorpiones increased to 23.91, 17.39, 13.04, 8.69, and 6.52%, respectively, whereas Carabidae decreased to 13.04%.

The results of mobile cages of transects A and B indicate that in April mean individuals/m$^3$ were 46.9, mean fresh weight of plants/m$^3$ was 4,889.06 g. This corresponded to 14.7 mean individuals/1,000 g of fresh plants. In October mean individuals/m$^3$ were 522.5, mean fresh weight of plants/m$^3$ was 4,468.9 g. This corresponded to 57.26 mean individuals/1,000 g of fresh plants. The results of mobile cages of transects C and D indicate that in April mean individuals/m$^3$ were 26.66, mean fresh weight of plants/m$^3$ was 2,139.6 g. This corresponded to 11.33 mean individuals/1,000 g of fresh plants. In October mean individuals/m$^3$ were 6.9, mean fresh weight of plants/m$^3$ was 1,693.66 g. This corresponded to 4.86 mean individuals/1,000 g of fresh plants.

During April, of the 86 coleopteran species sampled the most abundant belonged to two Staphylinidae (Table 1). Other species classified as abundant were species of the Chrysomelidae and Carabidae families. Only two species were found as influent and the rest of the 80 species were classified as recedent. In October, the picture slightly changes and of the 43 coleopteran species captured, only *Euaesthetus* sp. remained abundant (even if with a smaller percentage). In this case the most abundant species was a Cicindelidae. This was followed by a Carabidae, a Tenebrionidae, a Curculionidae, and a Nitidulidae. During autumn the number of influent species increased to 7 whereas the remaining 30 species were classified as recedent.

**TABLE 1**
**Abundant Species of the Coleoptera Found at El Bessila During the Two Seasons in All Transects**

| April | n = 3,954 | % | October | n = 598 | % |
|---|---|---|---|---|---|
| Staphylinidae | *Euaesthetus* sp. | 42.48 | Cicindelidae | *Cephalota litorea* | 22.91 |
| | *Hygronoma* sp. | 17.42 | Carabidae | *Agonum* sp. | 12.38 |
| Chrysomelidae | *Cryptocephalus* sp. | 11.40 | Tenebrionidae | *Blaps nitens nitens* | 12.21 |
| Carabidae | *Dicheirotrichus* sp. | 8.57 | Curculionidae | *Cleonus* sp. | 7.02 |
| | | | Staphylinidae | *Euaesthetus* sp. | 5.52 |
| | | | Nitidulidae | *Carpophilus* sp. | 5.18 |

The Isopoda belonged to three species: *Halophiloscia couchii*, *Porcellio* sp., and *Agabiformius obtusus.* In April (n = 1,410), *H. couchii* was the most abundant species (97.52%) whereas the other two species were collected and classified as recedent. In October (n = 311), *H. couchii* remained abundant (85.85%) together with *Porcellio* sp. (11.90%).

For the Coleoptera, the α diversity index was significantly higher in April than in October (Table 2). This was due to the presence of a double number of species in spring compared to autumn with 6.6 times more individuals. On the total, the α diversity index was 18.75. Analysing the isopod community, a higher α was obtained in October compared to April and this was in relation to the higher number of individuals found in that month. On the whole, the isopod community presented a quite low α value 0.353. The evenness index, estimated for the coleopterans and isopods over the entire system was 0.538 and 0.195, respectively, and in both cases this was higher in October than in April. Simpson's dominance index, calculated for the coleopterans and isopods as a total, was 0.184 and 0.911 and for both orders more dominant species were found in April than in October.

**TABLE 2**
**Ecological Indices of the Coleoptera and Isopoda Captured in April and October Calculated for All Transects and on the Total**

| | Coleoptera April | Coleoptera October | Coleoptera | Isopoda April | Isopoda October | Isopoda |
|---|---|---|---|---|---|---|
| **n** | 3,954 | 598 | 4,552 | 1,410 | 311 | 1,721 |
| **n species** | 86 | 43 | 103 | 3 | 3 | 3 |
| **Fisher et al.** | 15.51 | 10.62 | 18.75 | 0.363 | 0.460 | 0.353 |
| **Fisher's conf. lim.** | 1.28 | 1.81 | 1.41 | | | |
| **Brillouin** | 2.127 | 2.664 | 2.440 | 0.140 | 0.455 | 0.213 |
| **Brillouin max** | 4.392 | 3.607 | 4.539 | 1.093 | 1.080 | 1.094 |
| **Pielou-Brillouin** | 0.484 | 0.739 | 0.538 | 0.128 | 0.421 | 0.195 |
| **Simpson** | 0.233 | 0.100 | 0.184 | 0.951 | 0.751 | 0.911 |

## DISCUSSION

Species richness, abundance, composition, and distribution of terrestrial arthropod communities in tidal marshes are generally limited by ecological factors such as vegetation cover, tidal inundation, humidity, salinity, organic matter contents, etc. These arthropods are highly specialised and possess a set of adaptations to the extreme osmotic and microclimatic conditions of these habitats[23]. Tidal marshes are principally dominated by halophylic or halobiontic species[6], and contain elements of both terrestrial and marine communities[24]. The fauna of El Bessila was dominated by amphipods in both seasons on the seaward side of the islet, whereas it was dominated by Collembola on the landward side during spring. The amphipods belonged to species common on seashores, river mouths, and salt marshes with a wide geographical distribution in the Mediterranean and Black Sea. These were found in correspondence to the wrack mounds of *Zostera* sp. of zone 1 and were seen to escape the wracks and take refuge at the base of the vegetation with the advancing tides. The great numerical increase that occurred in autumn was in relation to the amphipod's life cycle. The second most consistent order was the Collembola that represent the main soil animal taxa in tidal salt marshes[8]. These mainly belonged to the Isotomatidae and Entomobrydae families and were more abundant in zone 2 in correspondence to the thicker vegetation. This agrees with Sterzynska and Ehrnsberger's[8] findings that reported a greater abundance of Collembola in older salt marsh habitats with a dense vegetation of *Salicornia* spp. than in younger ones with poor vegetation cover. In the vegetation clearings inland of El Bessila, ant colonies were extremely frequent together with a prominent beetle community. Samplings with mobile cages showed that in spring ground beetles were the most represented category found associated with the vegetation. This was due to the presence of nocturnal species that during the day rested under the vegetation cover.

Considering the results of macrofauna/plant ratio of the seaward transects, the great increase of individuals found in October was due to the massive presence of amphipods that in this season were in their reproductive phase. Instead in April the high number of individuals/m$^3$ in mobile cages of landward transects was principally due to the great amount of Carabidae found in this period. Thiele[25] showed that in salt marsh areas relatively high densities, up to 10 to 20 individuals/m$^2$, could be reached for ground beetles.

Beetles were more abundant in spring than in autumn months, but to this marked decrease in sample numbers corresponded an increase of the number of abundant species with the change of the season. Furthermore the spectrum of the beetle community completely changed from spring to autumn months. Of the Staphylinidae, *Euaesthetus* sp. was by far the most common genus found, especially in spring and together with *Hygronoma* sp. they made up 60% of the entire beetle community. Another quite abundant beetle during spring season was the Chrysomelidae *Cryptocephalus* sp. found on yellow composite flowers whereas the Carabidae *Dicheirotrichus* sp. was mainly found on the muddy and salty inland areas of the landward transects. In autumn the most abundant beetle species, *Cephalota litorea*, was common on moist clay soil and on barren spots and together with the carabid *Agonum* sp. a shore-dwelling species and the tenebrionid *Blaps nitens nitens* constituted about 50% of the total beetles sampled. In general higher species richness was found for the Carabidae family in both seasons. As a matter of fact, several ground beetles are highly specialised halobiontic species and limit their occurrence to one or several salt marsh microhabitats[25]. The second richest family, the Tenebrionidae, occurred with genera with wider habitat occupancy and ranged from typical psammophilic to halophilic species. Adam[26] suggested that many salt marsh arthropods show very wide distribution patterns in relation to their high dispersal capacity.

The Isopod community of El Bessila was dominated by *Halophiloscia couchii*, a terrestrial species with a wide geographical distribution from the Mediterranean Sea to the Atlantic coasts of Europe and Africa. Being a halophilic it can be found along coarse sandy shores, lagoons, and salt marsh areas. *Agabiformius obtusus,* a holomediterranean species, was captured in very low quantities and only in spring season. The third species, belonging to the *Porcellio* genus, is probably new to science and still must be described.

The overall results of the ecological indices showed that the beetle and isopod community were richer in spring than in autumn. For the beetles the diversity index was moderately high, whereas for the isopods it was extremely low. Salt marshes are generally considered low-diversity ecosystems, but this depends both on the considered groups as well as the spatial scale involved. Wiegert and Pomeroy[27] suggested that not more than 100 insect species might be expected because of the dearth of niches in a system with a few higher plants. At El Bessila this was not the case because beetles alone made up about 100 species in the two seasons. The relatively high diversity can be related to the presence of high tidal excursions that cast ashore a great amount of wracks. These could have been colonised by a conspicuous variety of phytophagous and scavenger species that might have successively attracted predaceous species. This was also confirmed by the values of $\alpha$ that decreased from the seaward face towards land. However for the landward transects an opposite trend was obtained showing the importance of the vegetation compared to the open areas of the pan. The $\alpha$ diversity index calculated for the isopod species was extremely low in relation to the presence of only three species of which one dominant.

As conclusion, the arthropod community of El Bessila showed variations both in space and in time according to the season and the area sampled. The high diversity found was related to the high tidal excursions that are probably one of the most important ecological factors modelling the structure of the community. The overall results show that El Bessila is a very special environment where appropriate conservation management measures are urgently needed.

## ACKNOWLEDGEMENTS

The study was funded by the European Commission, Programme INCO-DC, Project title “Bases for the Integrated Sustainable Management of Mediterranean Sensitive Coastal Ecosystems”, contract ERB-IC 18- CT-98-0270. The study complies with the current laws of Tunisia. We acknowledge the Faculté des Science de Tunis, the Agence pour la Protection et Amenagement du littoral, and the Triage des Forêts Graiba for their assistance in field activities. We would like to thank Dr. Stefano Taiti who identified the Isopoda species.
